# Variability in Insurance Coverage for Adult Voice Therapy in The United States of America

**DOI:** 10.1002/lary.70181

**Published:** 2025-10-15

**Authors:** Kevin B. Xiao, Carlye B. Goldenberg, Carson K. Gates, Mark R. Gilbert

**Affiliations:** ^1^ Department of Otolaryngology – Head and Neck Surgery University of Missouri Columbia Missouri USA; ^2^ University of Missouri School of Medicine Columbia Missouri USA; ^3^ Department of Otolaryngology – Head and Neck Surgery University of Illinois College of Medicine Chicago IL USA

**Keywords:** dysphonia, insurance, speech therapy, voice therapy

## Abstract

**Objectives:**

Voice therapy is often the first‐line treatment and an adjunct to medical or surgical management of dysphonia. However, a minority of patients with dysphonia utilize voice therapy. Insurance coverage can affect patients' decisions and ability to pursue voice therapy. The objective of this study was to investigate variability in insurance coverage of voice therapy for treatment of dysphonia in the United States of America.

**Methods:**

A cross‐sectional study was performed to review insurance policies from the top three commercial insurers and Medicaid in all 50 states and District of Columbia (DC). Top three commercial insurers were determined using 2021 enrollment data. Policies for commercial insurers and Medicaid in all 50 states and DC were then identified using internet search. Voice therapy coverage was categorized as covered, requiring prior authorization, determined on case‐by‐case basis, or not covered. Copay and visit limitations were also assessed.

**Results:**

Among 153 commercial insurers, 135 (88.2%) provided coverage for voice therapy. 39 (76.5%) state Medicaid plans provided coverage for voice therapy. There was no association between coverage status and insurance type (*p* = 0.06). Commercial insurers (34.09 ± 13.8) were associated with significantly higher annual visits limit than Medicaid (23.8 ± 10.1) (*p* = 0.02). There was a significant association between commercial insurers and documented copays (*p* = 0.01).

**Conclusions:**

While most of the commercial plans and Medicaid provide voice therapy coverage, discrepancies still exist, and many insurance providers do not provide coverage. This variability may contribute to disparities in access and outcomes for individuals with dysphonia.

**Level of Evidence:**

N/A.

## Graphical abstract

A cross‐sectional study was performed to review online health policy information related to the coverage of voice therapy among the three largest commercial health plans and Medicaid in each state and DC. Although most of the top commercial insurers and state Medicaid plans provided coverage for voice therapy, a large number still do not. Differences in annual visit limits and copays still exist as well among these insurance plans.

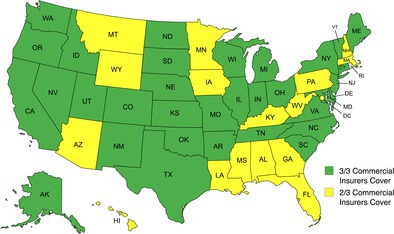

## 1 Introduction

Dysphonia is a common disorder that is estimated to affect one in 13 adults annually and 1 in five adults in their lifetime in the United States [1, 2]. Despite its typically benign etiology, dysphonia significantly impairs both voice‐related and overall quality of life. Bhattacharyya found that 85% of adults in the United States described their dysphonia as a problem [2]. Additionally, dysphonia incurs significant costs to both patients and the healthcare system [3, 4]. Dysphonia in adults has been associated with four additional days of lost work per year and contributes $5 billion to direct healthcare costs annually [3, 4, 5].

Voice therapy may be used as the primary treatment, an adjunct to medical or surgical management, a diagnostic tool in the treatment of dysphonia, or even a preventative treatment to preserve vocal health [6, 7]. Patients undergoing surgical intervention can still benefit from voice therapy as a way to maximize their long‐term post‐surgical voice [6]. Voice therapy has shown efficacy when used to treat a wide array of voice disorders including muscle tension dysphonia, benign vocal fold lesions, vocal fold atrophy, and neurologic disorders [5, 6, 7, 8].

Despite its efficacy, almost half of patients who are recommended voice therapy do not initiate treatment and 26%–47% of patients do not return after their initial evaluation [9, 10, 11]. In patients who do not initiate or adhere to voice therapy, insurance is commonly cited as a reason due to lack of coverage or the associated costs [5, 9]. While insurance is known to be a major barrier to care in the treatment of dysphonia, no studies have investigated variability in insurance coverage of voice therapy for the treatment of dysphonia in the United States. The purpose of this study is to investigate the variability in insurance coverage among the top commercial health plans and Medicaid within the United States.

## 2 Materials and Methods

### 2.1 Institutional Review Board Approval

This study was approved by the Institutional Review Board at the University of Missouri and was granted exemption (IRB #2116066).

### 2.2 Study Design

The top three commercial insurers from each state and Washington, DC, were identified using enrollment data from the large group market of people enrolled in comprehensive major medical insurance and mini‐med health plans from 2021 [12]. A cross‐sectional review was performed to evaluate a total of 153 commercial health plans and 51 Medicaid health plans for a total of 204 health plans. The primary outcome was coverage status for voice therapy in adults. Secondary outcomes included documented copays in health plans and limits on the number of annual therapy visits.

### 2.3 Data Collection

A Google web search was conducted in October 2024 to identify health plan policies for each commercial insurer and Medicaid. The web search was performed by authors K.B.X, C.K.G, and C.B.G. and was performed using the following search terms: “speech therapy”, “voice therapy”, “dysphonia”, “92507”, and “R49.0,” in addition to either the state Medicaid (e.g., “Alabama Medicaid”) or commercial insurer name (e.g., “Alabama Blue Cross Blue Shield”). The most recent policies identified were reviewed. Health plan policies gathered from the web search were reviewed by K.B.X prior to assessment. Final assessment of coverage status was performed by K.B.X. When available, CPT code 92507 and ICD‐10 code R49.0 were used to identify specific coverage of voice therapy. CPT code 92507 includes the individual treatment of speech, language, voice, communication, and/or auditory processing disorders. ICD‐10 code R49.0 includes the diagnoses of dysphonia and hoarseness. When these codes were not available, the policy was interpreted based on the language used to describe coverage status for various adult services provided by outpatient speech therapy. If the policy was not specific in its distinction of the various services provided by speech therapy, coverage status was determined based on the overall coverage status of outpatient adult speech therapy services.

Policies were reviewed and categorized as covered, requiring prior authorization, determined on a case‐by‐case basis, or not covered. Policies were categorized based on their description for coverage of CPT code 92507 or ICD‐10 code R49.0 or provided descriptions of what types of conditions or services were covered. Examples of these include documentation of coverage for benign vocal fold lesions, treatment of resonance, and treatment of voice quality, pitch, and respiration. In some cases, coverage was granted based on the requirements of medical necessity to obtain coverage. These criteria included but were not limited to documentation of a diagnosis of a voice disorder by a physician, documentation of a physician recommendation for speech therapy, therapy be performed on an outpatient individual basis, therapy be performed by a speech language pathologist (SLP), expectation of improvement with therapy, documentation of goals of therapy. In these cases, we considered voice therapy to fit these criteria, and we used the coverage policy listed for these criteria. In some cases, policies indicated all speech therapy services were covered and coverage of voice therapy was included in these cases. Age limitations were occasionally documented with different policies for patients younger or older than 21 years old. Policies for patients under 21 primarily addressed developmental delays. Assessment of coverage status focused on those for patients older than 21, whose needs better aligned with voice therapy indications.

Policies were also reviewed to determine if a copay was required with visits or if there were limitations on the number of annual visits with speech therapy. Copay documentation was variable, with some companies providing information about pricing while others only listed that a copay was required. Copays were categorized as either reported in the policy or not reported. The number of annual visits was reported as a numerical value.

### 2.4 Data Analysis

Statistical analysis was performed using IBM SPSS Statistics for Macintosh, Version 29.0 (Released 2023; IBM Corp., Armonk, New York, USA). An alpha value of 0.05 was considered statistically significant for all tests. Chi‐squared analysis was used to assess associations between insurer type and coverage status, documented copays and insurer type, and documented copays and coverage status. Student's two‐tailed t‐test was used to assess associations with visit limits on speech therapy visits and insurance type.

## 3 Results

The top three commercial insurers in each state and the District of Columbia (DC) based on enrollment data from 2021 were identified, resulting in a review of 153 commercial health plan policies [12]. These commercial insurers accounted for between 45% and 100% of the total market share of people enrolled in large group commercial health insurance in each state. A total of 51 Medicaid health plan policies from each state and DC were also reviewed, resulting in a total of 204 health plan policies reviewed in this study. Policies detailing coverage of voice therapy or speech therapy were identified for all 204 health plans through an online web search. CPT code 92507 and/or ICD‐10 R49.0 was used to help identify coverage status in 82 (40.2%) of all health plans.

**FIGURE 1:**
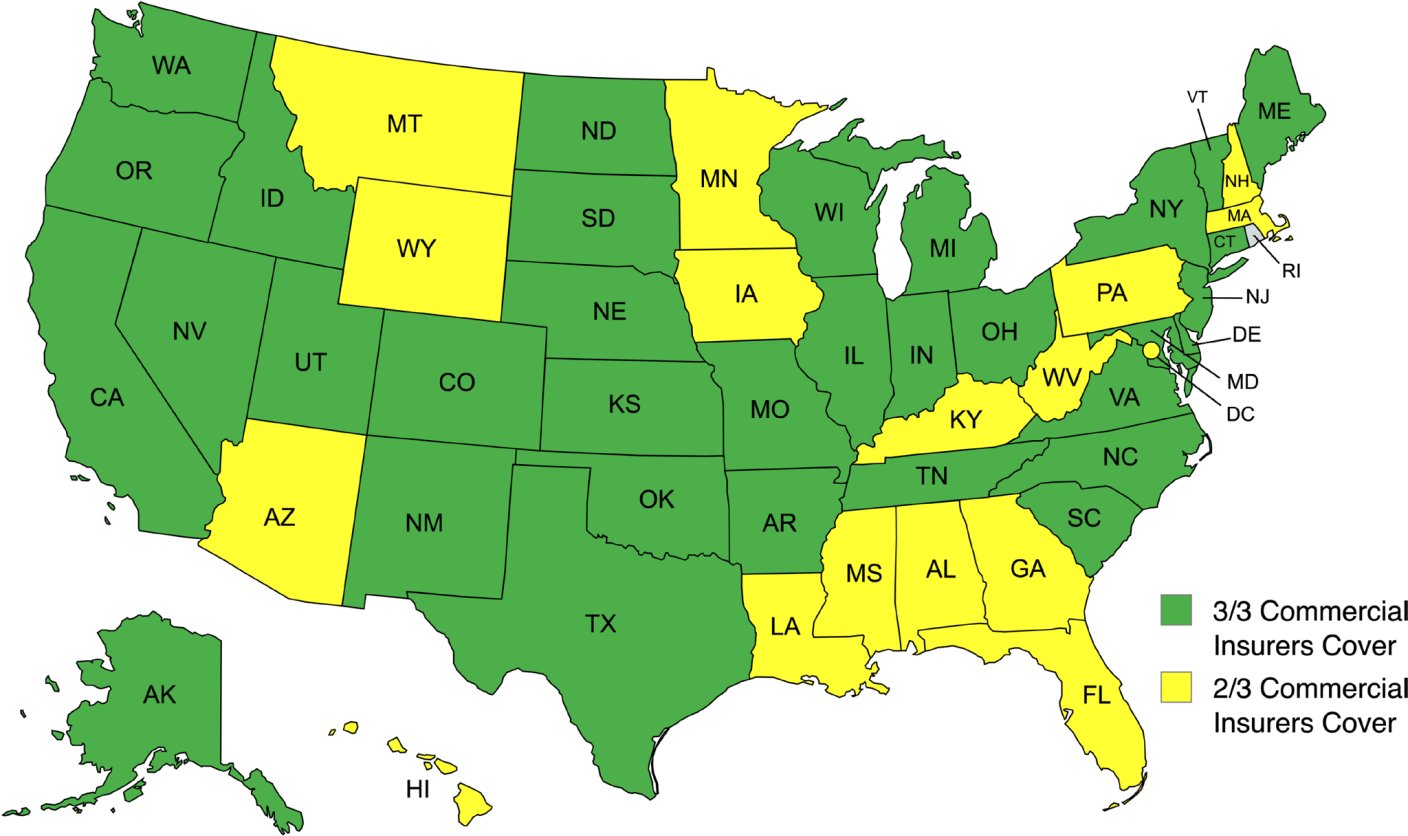
Commercial insurance coverage of voice therapy in the United States; 34 (66.7%) states documented coverage for voice therapy among all three of the top commercial insurers. Image generated with mapchart.net.

**FIGURE 2:**
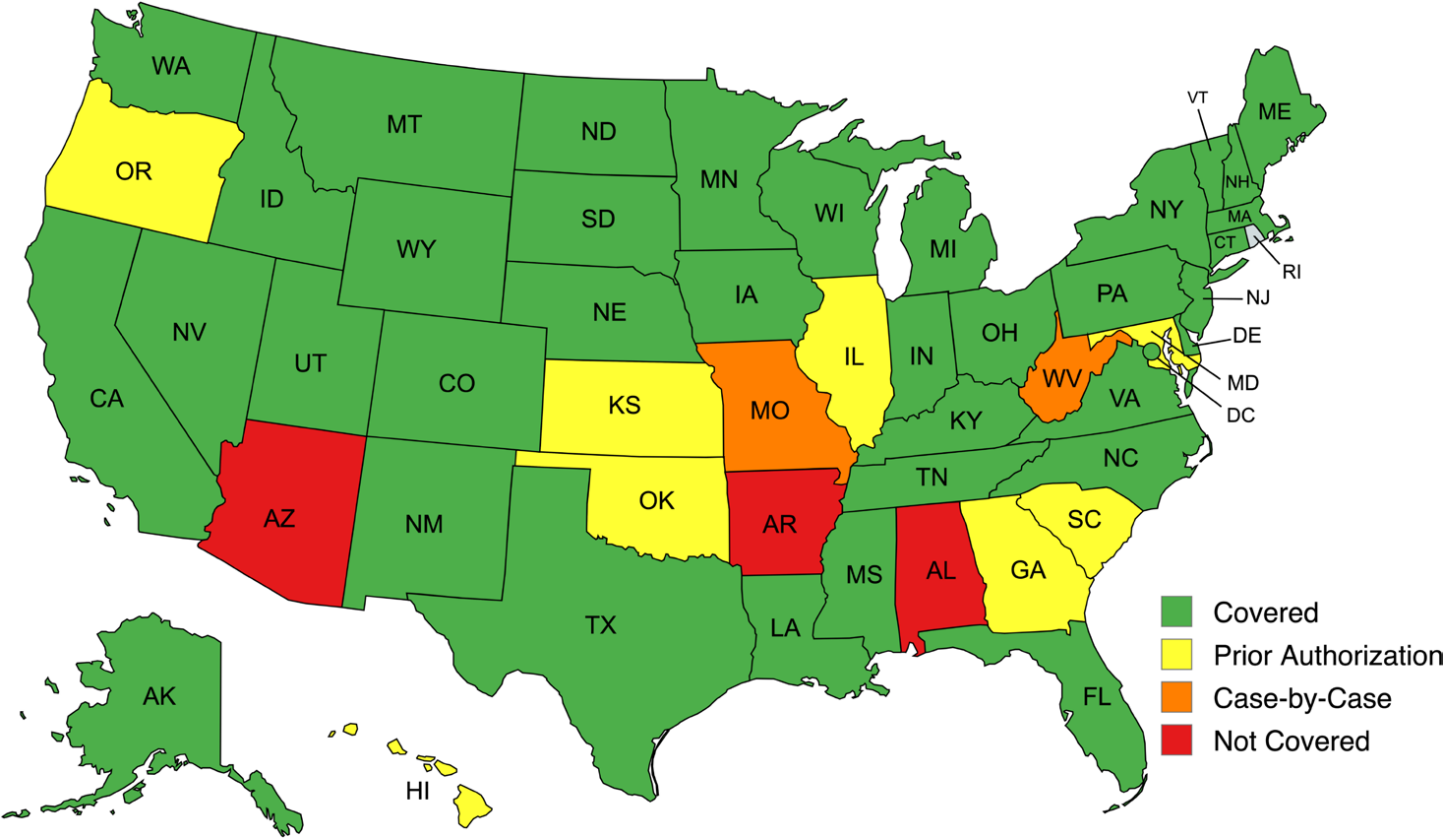
Medicaid coverage of voice therapy in the United States; 39 (76.5%) state Medicaid plans including DC documented coverage for voice therapy. Image generated with mapchart.net.

Overall, 135 (88.2%) of commercial insurers and 39 (76.5%) of state Medicaid provided coverage for voice therapy (Table 1). There was no significant association between the coverage status of voice therapy and insurer type on the chi‐square test of independence [*X* ^2^ (3, *N* = 204) = 7.3, *p* = 0.06]. Three out of three of the top commercial insurers provided coverage in 34 (66.7%) states, and two out of the top three commercial insurers provided coverage in the remaining 17 (33.3%) states, including DC (Figure 1). Medicaid coverage status across each state is shown in Figure 2.

**TABLE 1:** Voice therapy coverage status by insurer type.

		Commercial	Medicaid	Total	*p*
Coverage status	Covered	135	39	174	0.06
	Prior authorization	14	7	21	
	Case‐by‐case	3	2	5	
	Not covered	1	3	4	
Total		153	51	204	

Limitations on the number of visits allowed for speech therapy services were documented by 34.3% (*n* = 70) of all reviewed health plans. When these limits were reached, the specific policy varied and sometimes required prior authorization for further visits or provided no further coverage. Policy limitations on visits applied to either speech therapy visits alone or applied to the total of all outpatient therapy services including physical, occupational, and speech therapy. The overall average limit on the number of visits was 32.3 ± 13.7 visits. When reported, commercial insurers limited speech therapy visits to an average of 34.09 ± 13.8 visits while Medicaid limited speech therapy visits to an average of 23.8 ± 10.1 visits. Commercial insurers allowed for significantly more annual visits (mean difference = 10.33; 95% CI, 1.9–18.7; *p* = 0.02) compared to Medicaid.

Documentation of a copay with visits was found in 42 (20.6%) health plans. Copays were documented on 38 (24.8%) of commercial health plans and four (7.8%) Medicaid plans. There was a significant association between commercial insurers and a documented copay on chi‐square test of independence [*X* ^2^ (1, *N* = 204) = 6.8, *p* = 0.01]. There was no significant association between coverage status and documented copays [*X* ^2^ (3, *N* = 204) = 2.5, *p* = 0.47].

## 4 Discussion

This study investigated the coverage of voice therapy by the top three commercial insurers within the large group market and Medicaid in each state and DC. While this study does not capture all patients with commercial health insurance, total enrollment among these top three commercial insurers in the large group market accounted for an estimated 40,944,759 individuals and for 45% to 100% of the total market share of people enrolled in large group commercial health insurance in each state and DC in 2021 [12]. In August 2024, approximately one in six adults was enrolled in Medicaid [13]. Overall, this study examines policies related to voice therapy coverage that affect a large population of insured Americans.

Insurance coverage has been cited in some studies as a barrier for not initiating or adhering to voice therapy [5, 9, 10, 14]. We found that most health plans provided coverage for adult voice therapy, with 135 (88.2%) commercial health plans and 39 (76.5%) Medicaid plans providing coverage. Only one commercial health plan and three Medicaid plans documented no coverage for voice therapy services. Despite the high rates of documented coverage, prior studies have reported substantial nonadherence, often attributed to perceived or actual lack of coverage. Portone et al. [9] reported in their study an initial voice therapy attendance rate of 61.6% and a 53.4% follow‐up rate. They reported that 48% of patients who were surveyed regarding their nonadherence cited insurance denials as their primary reason for nonadherence to voice therapy recommendations [9]. This discrepancy may reflect some of the policies where prior authorization is required or determines coverage on a case‐by‐case basis, where actual access to services may be uncertain or delayed. Coverage is not necessarily guaranteed in these policies.

Although 39 state Medicaid plans documented coverage, studies have demonstrated that patients with public insurance were more likely to delay care or not attend voice therapy compared to those with commercial insurance [10, 14]. Patients with private insurance had 2.35 times the odds of attending voice therapy compared to those with public insurance [10]. There was no association between coverage status and type of insurance in our study. This lack of association may be reflective of other socioeconomic factors, as patients on public insurance like Medicaid are typically of lower socioeconomic status and have other social determinants of health that affect their ability to receive care. Hur et al. [14] demonstrated that patients from racial minority groups, low‐income backgrounds, or those with public insurance such as Medicaid were more likely to delay care due to reasons such as lack of transportation to visits.

In addition to coverage status, copays may also affect patients' decision to attend voice therapy. Misono et al. [15] reported that 62% of patients who were recommended to undergo voice therapy cited the copay fee as a reason to pursue therapy. Documentation of copay with visits was found in 38 (24.8%) commercial health plans and four (7.8%) Medicaid plans, and there was a significant association between commercial insurers documenting a copay with visits in their policies. This association is congruent with prior studies which demonstrated patients with commercial insurance were more likely to attend voice therapy. However, it is unclear as to why this relationship promotes voice therapy attendance.

Patients typically undergo multiple voice therapy sessions over several weeks, with North American patients undergoing an average of 12.52 sessions over 7.62 weeks for 12.15 h [16]. The number of voice therapy sessions that patients undergo depends on a variety of factors, including the etiology of dysphonia, the severity of their dysphonia, and the goals of treatment [6, 7, 16]. Fujiki et al. reported an average of 4.3 sessions for presbyphonia to 6.7 sessions for benign vocal fold lesions, with an overall average of 5.3 sessions prior to discharge from therapy. It is important to know how many sessions a patient may require, given insurance limitations placed on the number of annual visits. Additionally, it is important to consider that up to 14.5% of patients may return for additional voice therapy after their initial discharge [7]. In our study, 70 (34.3%) health plans documented limitations on annual visits with speech therapy, with an average of 32.3 ± 13.7 annual visits. Some plans allowed as few as five annual visits, while others allowed up to 90 annual visits. Commercial insurers (34.09 ± 13.8) allowed for significantly more annual visits compared to Medicaid (23.8 ± 10.1 visits). When this limit was reached, policies sometimes required prior authorization for further visits or did not cover any further visits. Often, the annual visit limitation included other outpatient therapy services such as occupational and physical therapy. This is an important detail to consider when recommending voice therapy for patients, as those with other therapy needs may not have sufficient remaining visits for an adequate course of voice therapy. Overall, the limits on annual speech therapy visits demonstrated in this study should be sufficient for patients to cover the average number of therapy sessions required for their dysphonia.

This study has several limitations related to its design. It is difficult to truly obtain an accurate up‐to‐date representation of insurance coverage for voice therapy given the vast heterogeneity of insurance. We used enrollment data from 2021 to identify which commercial insurance health plans to review, and these may be outdated. We also chose to focus on the large group market given the larger enrollment compared to the small group market and the individual market, and this does exclude a portion of the population [12, 17, 18]. Even within large group markets, there may exist a variety of plans from health maintenance organizations to preferred provider organizations with differing coverage based on the tier within those plans. Interpretation of policy information was limited to the information available through online web search. Some of these data may be outdated, incomplete, and were often vague, and may not truly reflect accurate coverage of voice therapy services and information related to visit limits or copays. Additionally, we did not include Medicare in this study as its policies are generally standardized, and it typically covers medically necessary outpatient speech therapy services if it is certified by a physician or health care provider [19].

Future studies may investigate the relationship between coverage status of voice therapy across the United States on a state‐by‐state or national level and its impacts on patient outcomes, treatment adherence, and provider decision‐making.

## 5 Conclusion

While most of the top commercial health plans and Medicaid provide coverage for voice therapy, discrepancies still exist, and many insurance providers do not provide coverage. These variations may contribute to disparities in access to treatment for individuals with dysphonia. Efforts to improve policy transparency and expand consistent coverage could support timely and equitable access to voice therapy and ultimately improve patient outcomes.

## Conflicts of Interest

The authors declare no conflicts of interest.

## Data Availability

The data that support the findings of this study are available from the corresponding author upon reasonable request.
